# Altered Expression of Complement Regulatory Proteins CD35, CD46, CD55, and CD59 on Leukocyte Subsets in Individuals Suffering From Coronary Artery Disease

**DOI:** 10.3389/fimmu.2019.02072

**Published:** 2019-08-29

**Authors:** Nitesh Mishra, Madhav Mohata, Rajeev Narang, R. Lakshmy, Anjali Hazarika, R. M. Pandey, Nibhriti Das, Kalpana Luthra

**Affiliations:** ^1^Department of Biochemistry, All India Institute of Medical Sciences, New Delhi, India; ^2^Department of Cardiology, All India Institute of Medical Sciences, New Delhi, India; ^3^Department of Cardiac Biochemistry, All India Institute of Medical Sciences, New Delhi, India; ^4^Blood Bank, Cardio-Neuro Centre, All India Institute of Medical Sciences, New Delhi, India; ^5^Department of Biostatistics, All India Institute of Medical Sciences, New Delhi, India

**Keywords:** Cregs, complement, coronary artery disease, inflammation, granulocytes

## Abstract

Studies conducted in animal models have suggested that membrane complement regulatory proteins play an important role in the pathophysiology of coronary artery disease (CAD). In this study, a total of 100 individuals, with stable CAD and 100 healthy controls, both groups predominantly male, were recruited. We evaluated the plasma levels of complement regulatory proteins (Cregs) CD35, CD46, CD55, and CD59 and their surface expression on granulocytes, lymphocytes, and monocytes by flow cytometry. The mRNA expression of these Cregs in total leukocytes was determined by quantitative PCR. The soluble forms of Cregs, C3c, Mannose binding protein-associated serine protease 2 (MASP-2), Platelet activating factor-acetyl hydrolase (PAF-AH), and inflammatory cytokines were quantified by ELISA. High plasma levels of C3c, indicative of complement activation, in addition to significantly low levels of Cregs, were observed in CAD patients. A significantly lower expression of CD46 and CD55 on the surface of lymphocytes, monocytes, and granulocytes and higher surface expression of CD35 and CD59 on granulocytes (*p* < 0.0001) was seen in CAD patients as compared to healthy donors. The high expression of CD59 on granulocytes positively correlated with the severity of disease and may serve as a potential marker of disease progression in CAD.

## Introduction

According to the global burden of diseases 2015, coronary artery disease (CAD) is one of the leading causes of death globally, with the highest rates observed in central Asia and Europe (1). Atherosclerosis, characterized by the plaques of oxidized cholesterol-rich low-density lipoprotein (LDL) particles, fat and foam cells on the arterial wall, is the underlying cause of CAD (2). The last decade has seen a significant shift in the understanding of atherogenesis and inflammation (3–5). The complement system has been shown to be one of the determinants influencing the stability and rupture of atherosclerotic plaques (4). Further, the complement cascade plays an important role in CAD by promoting inflammation and by interacting with blood coagulation (6). Besides its traditionally well-known proinflammatory effects, complement activation is also necessary for clearance of immune complexes, debris, and apoptotic cells and may, therefore, exert dual–proatherogenic and atheroprotective–effects within the vessel wall (7).

The balance between activation and inhibition of the complement system is critical in controlling the level of inflammation generated, thus keeping potential self-harming inflammation under control (7, 8). Complement activation is finely regulated by complement regulatory proteins (Cregs). The Cregs, both soluble and membrane bound, are key molecules that regulate activation of the complement cascade, mostly at the C3 convertase step. Membrane-associated Cregs include complement receptor type 1 (CR1, CD35), membrane cofactor protein (MCP, CD46), decay-accelerating factor (DAF, CD55), and membrane-inhibitor of reactive lysis (MIRL, CD59), with each protein differing in its mechanism of action to regulate the complement cascade. The CD46 protein regulates C3 activation by functioning as a cofactor protein for factor I-mediated cleavage of C3b whereas CD55 inhibits the activation of C3 and C5, by preventing the formation of new and accelerating the decay of preformed C3 and C5 convertases. The CD35 protein shows both CD46 and CD55-like activities and additionally plays a role in immune complex processing and clearance by acting as a major immune adherence receptor. The CD59 protein acts at the terminal step of complement activation cascade and prevents the formation of the membrane attack complex (MAC) (7–9). Soluble complement regulators include factor H, factor I, C4b binding protein (C4BP), and C1 inhibitor (C1INH), that prevent the formation of convertase complexes, destabilize existing ones and enable the degradation of complement proteins C3b and/or C4b (10, 11). The soluble forms of CD35, CD46, CD55, and CD59 have been implicated as biomarkers for disease activity (3, 12–14).

Regulation of the balance between activation and inhibition of the complement system is critical in controlling inflammation and thus prevents self-damage caused by uncontrolled inflammation. In addition to their traditional role in modulating complement activation, several recent studies have shown novel functions for Cregs in modulating adaptive immunity and their influence on the pathophysiology of chronic inflammatory diseases (7, 14, 15). Dysregulation of Cregs, responsible for maintaining the homeostasis of the complement cascade, has been well-established in autoimmune disorders like rheumatoid arthritis (RA), Systemic Lupus Erythematous (SLE), Multiple Sclerosis (MS), bullous pemphigoid and in diabetic individuals with micro and macrovascular disorders (3, 15–18). The role of Cregs as markers of disease activity and therapeutic targets is being realized, though most of the existing studies are focused on autoimmune disorders and cancers (18–20). Complement proteins have been shown to play an important role in the pathophysiology of CAD (2) and in the control of inflammation in experimental animal models (21–23). However, limited information is available on the role of Cregs in CAD. Complement proteins have a strong costimulatory effect on B cells; complement receptors are expressed on all immune cells and have an important role in the development of T cell immunity (2, 7). Any alteration in the regulation complement activation can thereby lead to its dysregulation, and ultimately immune disorders.

The present study was aimed at the evaluation of complement activation, regulation, and inflammatory mediators, to gain an insight into the overall dynamics and alterations of Cregs in CAD patients with varying disease severity. Hundred healthy individuals and 100 CAD patients were recruited and evaluated for the surface protein and mRNA expression along with soluble levels of Cregs.

## Materials and Methods

### Study Participants

This study was conducted in subjects residing in North India. A total of 100 healthy donors, between the age of 45–55 years, visiting the Blood Bank, Cardiovascular and Neurosciences Center, and 100 individuals suffering from stable CAD visiting the Department of Cardiology, All India Institute of Medical Sciences, New Delhi, India were recruited for the study after obtaining written informed consent. Six milliliters venous blood was drawn in EDTA tubes from all the subjects. One milliliter of whole blood was used immediately for total RNA extraction and 100 μl for flow cytometric analysis of the surface expression of Cregs. The rest of the blood was processed to separate plasma by centrifuging at 1,000 × g for 5 min. The plasma samples were stored at −80°C for the measurement of soluble Cregs, C3c and inflammatory molecules. This study was reviewed and approved by the institute ethics committee (IECPG/306/27.04.2016).

### Immunofluorescence Staining for Flow Cytometry

The surface expression of CD35, CD46, CD55, and CD59 on subsets of leukocytes was assessed by flow cytometry. Briefly, 100 μl of blood was taken in each flow tube. Flurochrome tagged monoclonal antibodies [20 μl of PE—CD35, APC–CD55, FITC—CD59 (BD Biosciences, USA) and 5 μl of PE-Cy7—CD46 (Biolegend, USA)] were added. The tube was vortexed gently and incubated for 30 min at room temperature. After incubation, 2 ml of FACSlyse solution (BD Biosciences, USA) was added and incubated for 15 min. The tube was centrifuged at 600 × g for 10 min and the supernatant was discarded. To the pellet, 1 ml of PBS was added and the tube was again centrifuged at 600 × g for 10 min. The supernatant was aspirated and the cells were resuspended in 100 μl of paraformaldehyde and acquired on BD FACSCanto II (BD Biosciences). FlowJo V10.2 (Tree Star) software was used for setting the gating parameters and analysis of data. Median fluorescence intensity of CD35, CD46, CD55, and CD59 was calculated on leukocyte subsets of lymphocytes, monocytes, and granulocytes and were log-transformed for further analysis.

### Reverse Transcription and Quantitative PCR

Total RNA was isolated from 1 ml of whole blood using TRIzol reagent and then reverse transcribed using random hexamers. The reverse transcription was carried out in a thermal cycler at 25°C for 10 min, 42°C for 60 min and the reaction was stopped by incubating the RT-mixture at 70°C for 10 min to inactivate the Reverse Transcriptase enzyme. The RT-mixture containing cDNA was stored at −20°C and was used for downstream PCR amplifications. A negative control reverse transcription reaction was also performed without adding reverse transcriptase in the RT-mixture. Quantitative PCR (qPCR) was performed using SYBR Green I in BioRad i-Cycler (BioRad, Hercules, CA) with specific primers (Table 1) targeting different transcripts. The expected qPCR products were confirmed by performing a melt curve analysis and electrophoresis on a 1.5% agarose gel. GAPDH was used as the normalizing gene and ΔΔCt values for CD35, CD46, CD55, and CD59 were calculated, and log transformed for further analysis.

**Table 1:** Sequence, amplicon size, and melting temperature for each set of primers used for quantitative PCR of CD35, CD46, CD55, and CD59. GAPDH was used as the normalizing gene.

**Gene**	**Forward primer (5^**′**^-3^**′**^)**	**Reverse primer (5^**′**^-3^**′**^)**	**Tm**	**Amplicon size**
GAPDH	CTCCTGTTCGACAGTCAGCC	TGGAATTTGCCATGGGTGGA	58.4	235
CD35	GGACTGGTGCTAAGGACAGG	ATGATGCATGTGGCAGACGA	61.3	166
CD46	TTGCCATAGGAAAGCAGATGGT	GCTAAGCCACAGTTGCACTC	60.5	187
CD55	CCACCACACCAAATGCTCAAG	CCCTCCAAGAACTGGAGTGAC	61.2	218
CD59	CTGGAAGAGGATCTTGGGCG	AGGACAGACCCTCCTTGGAT	60.5	232

### Sandwich ELISA

Plasma levels of IL-4, IL-6, IL-8, IL-10, IFN-γ, soluble forms of CD35, CD46, CD55, and CD59, Mannose binding protein-associated serine protease 2 (MASP-2), Platelet activating factor-acetyl hydrolase (PAF-AH) and C3c were measured by commercially available sandwich ELISA kits (Hycult Biotech, Uden Netherlands & R&D Systems Inc. Minneapolis, USA). The samples were processed as per the manufacturers' protocol and the final concentrations of above molecules were estimated by computer-based curve fitting software (GraphPad Prism 6) using a 4-parameter logistic curve-fitting algorithm.

### Statistical Analysis

For a cross sectional comparison of two groups, a non-parametric Mann-Whitney U test (2-tailed) was used. For comparison of three or more groups, Kruskal-Wallis test was used. All statistical analyses were done using GraphPad Prism 6 software, USA. In all cases, *p*-values <0.05 were considered as significant. Data is represented as median with interquartile range and *p*-value is shown by asterisks with ^*^ = *p* < 0.05, ^**^ = *p* < 0.01; ^***^ = *p* < 0.001, and ^****^ = *p* < 0.0001.

## Results

### Patient Demographics

A total of 200 individuals were recruited for this study after obtaining written informed consent; 100 individuals diagnosed with CAD were recruited and were further subcategorized based on their vessel blockage. CAD patients with at least 50% stenosis of one, two and three of the major epicardial coronary arteries were classified into single vessel disease (SVD), double vessel disease (DVD), and triple vessel disease (TVD), respectively. Out of the 100 CAD patients, 67 had SVD, 20 had DVD, and TVD was seen in 13 individuals. All the CAD patients were on statin therapy (with majority on Atorvastatin and remaining few on Rosuvastatin). The statin treatment regimen was similar for CAD patients, regardless of the vessel status. Hundred age and sex matched healthy individuals with no history of heart disease were included as controls. None of the healthy controls were on statin therapy. The demographic profile of CAD patients and healthy donors is summarized in Table 2.

**Table 2:** Demographic and clinical profile of healthy individuals and coronary artery disease patients.

**Characteristics**	**Healthy donors (*n* = 100)**	**CAD (*n* = 100)**
Age	52.71 ± 8.63	56 ± 11
Sex (M/F)	(99/1)	(98/2)
Total cholesterol	150 ± 32.29	144.54 ± 33.65
Triglycerides	168 ± 63	183 ± 84
VLDL	38.1 ± 21.3	34.2 ± 16.9
LDL	75 ± 26	71 ± 23
HDL	41 ± 8.5	37 ± 7.5

### Differential Expression of Cregs on Leukocytic Subsets in CAD Patients and Healthy Individuals

**Figure 1:**
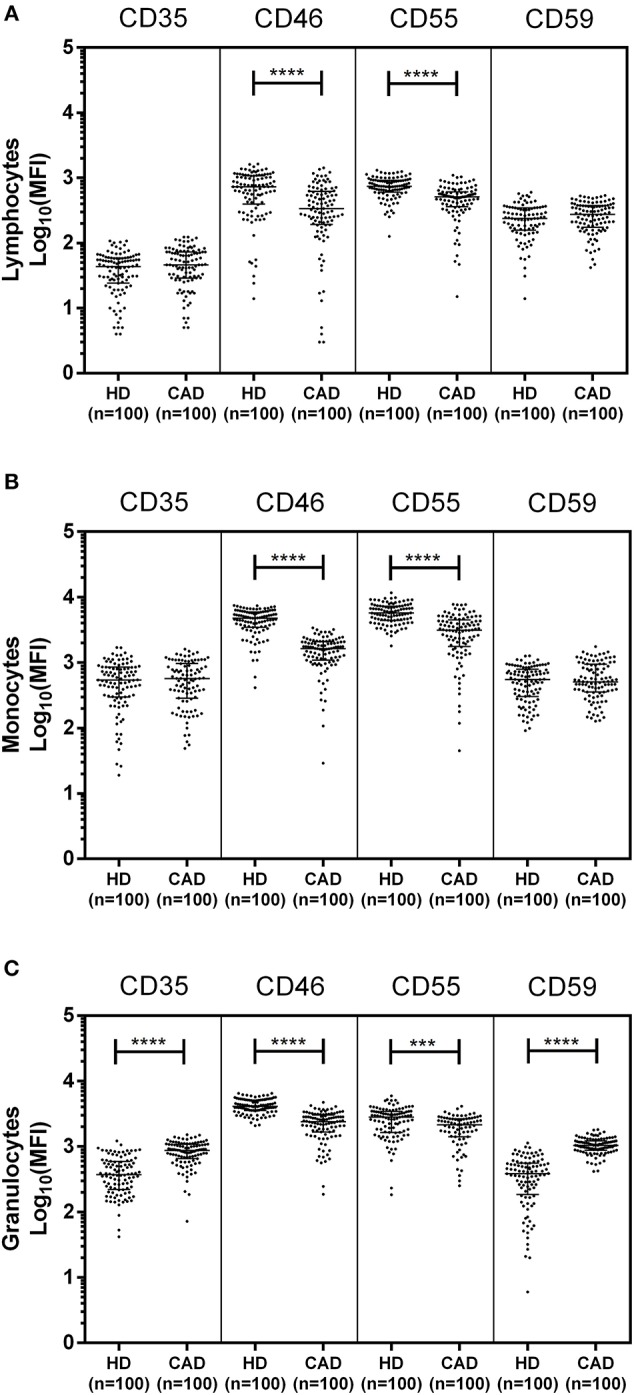
Differential surface expression of complement regulatory proteins on leukocytic subsets of coronary artery disease patients. Surface expression of CD35, CD46, CD55, and CD59 on lymphocytes, monocytes, and granulocytes of healthy donors, and coronary artery disease patients was assessed by flow cytometry. Comparison of log-transformed median fluorescence intensity (Log_10_MFI) of CD35, CD46, CD55, and CD59 shows significantly lower surface expression of CD46, and CD55 on all leukocytic subsets of CAD patients **(A–C)**. CD35 and CD59 surface expression was significantly higher on granulocytes of CAD patients **(C)**. Mann-Whitney *U* test was used for comparison of values between groups. Three asterisks (***) indicate a *p*-value of <0.001, and four asterisks (****) indicate a *p*-value of <0.001.

**Figure 2:**
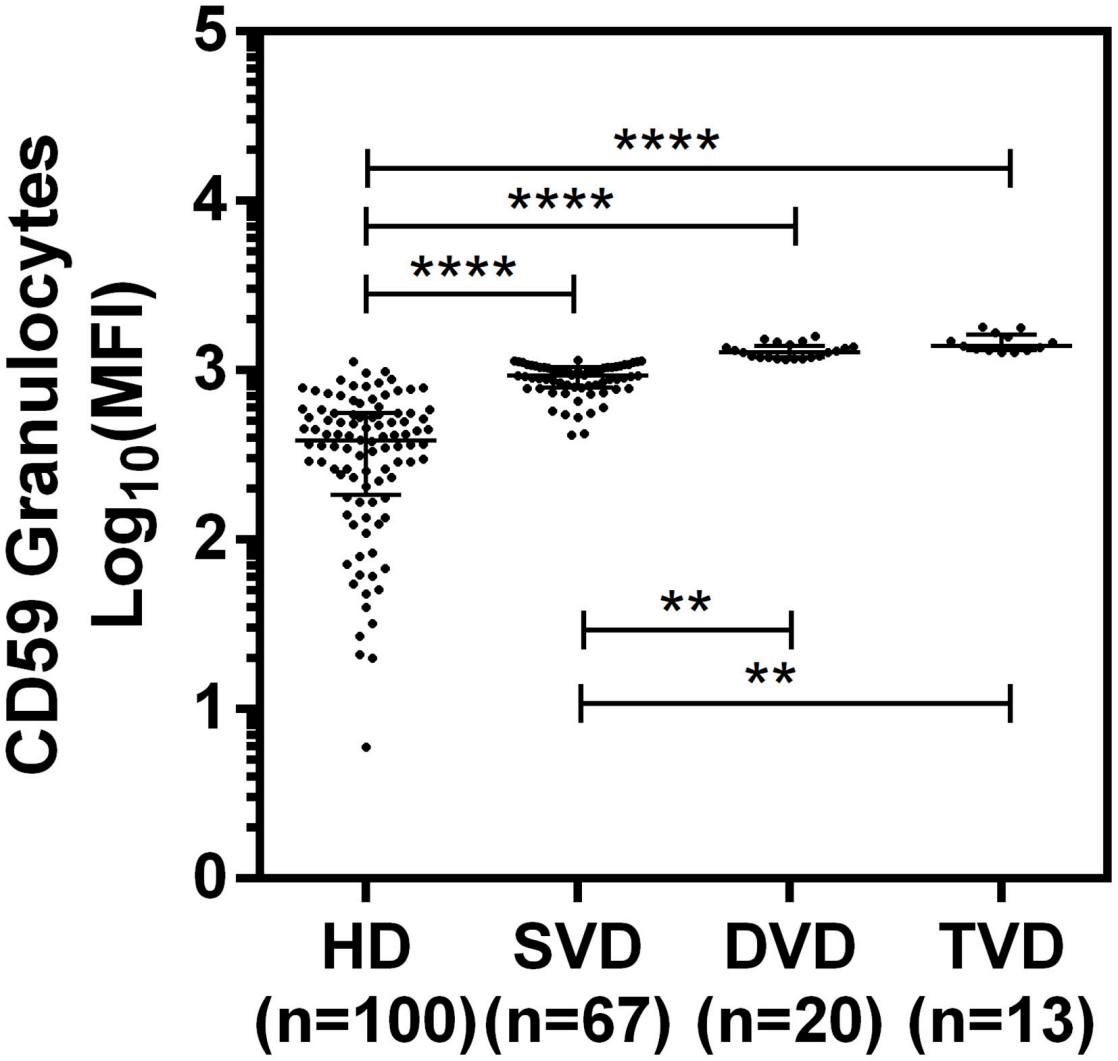
Differential surface expression of CD59 on granulocytes of coronary artery disease patients with varied vessel blockage. Comparison of log-transformed median fluorescence intensity (Log_10_MFI) of CD59 shows significantly higher surface expression on granulocytes with increasing severity of disease. The Mann-Whitney *U* test was used for comparison of values between two groups and Kruskal-Wallis test was used for comparison between three groups. Two asterisks (**) indicate a *p*-value of <0.01, and four asterisks (****) indicate a *p*-value of <0.001.

The surface expression of CD35, CD46, CD55, and CD59 on lymphocytes, monocytes, and granulocytes of the healthy donors and CAD patients was assessed. We observed a significantly lower expression of CD46 and CD55 on the surface of lymphocytes, monocytes and granulocytes and higher surface expression of CD35 and CD59 on granulocytes (*p* < 0.0001) in CAD patients compared to healthy donors (Figure 1). No significant difference was observed for the surface expression of CD46 and CD55 on all subsets of leukocytes within the CAD patients with different disease stages (data not shown). The surface expression of CD59 on granulocytes was significantly higher in individuals with multiple vessel blockage than in healthy donors with the highest expression observed in patients with TVD (Figure 2).

### Relative mRNA Expression of CD35, CD46, CD55, and CD59 in CAD Patients

**Figure 3:**
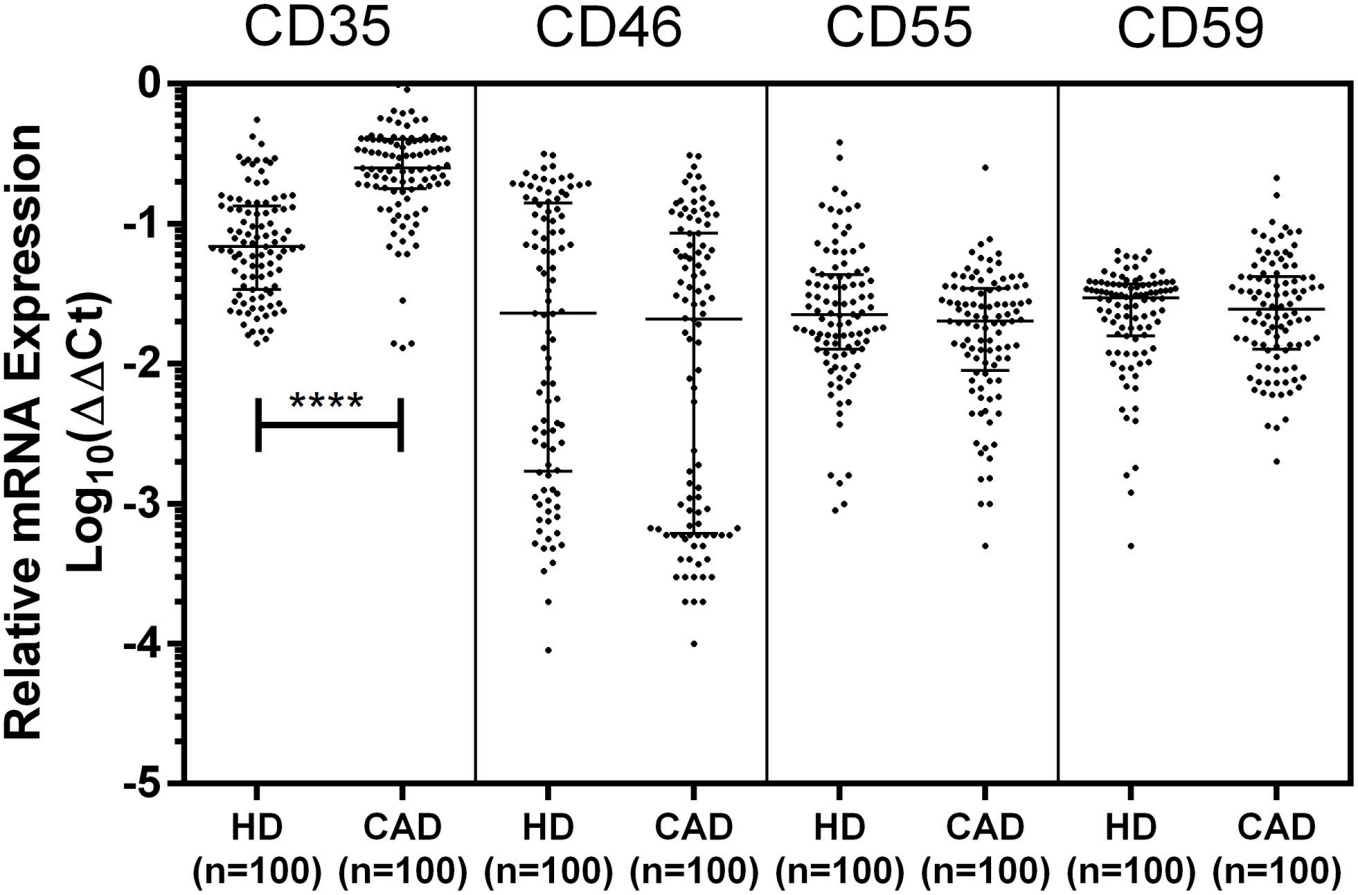
Relative mRNA expression of complement regulatory proteins in coronary artery disease patients. Relative mRNA expression of CD35, CD46, CD55, and CD59 showed higher mRNA expression of CD35 in CAD patients. ΔΔCt values were converted to logarithmic equivalents. Mann-Whitney *U* test was used for comparison of values between groups. Four asterisks (****) indicate a *p*-value of <0.001.

The mRNA expression of CD35, CD46, CD55, and CD59 was assessed by quantitative PCR using gene specific primers (Table 1). The mRNA expression of CD46, CD55 and CD59 did not significantly vary between the study groups. A significantly higher mRNA expression of CD35 was observed in the CAD patients than healthy donors (*p* < 0.0001) (Figure 3). The CD35 expression was comparable between CAD patients with different disease stages (data not shown).

### Plasma Levels of Soluble Complement Regulatory Proteins and Cytokines in Patients With Stable CAD

**Figure 4:**
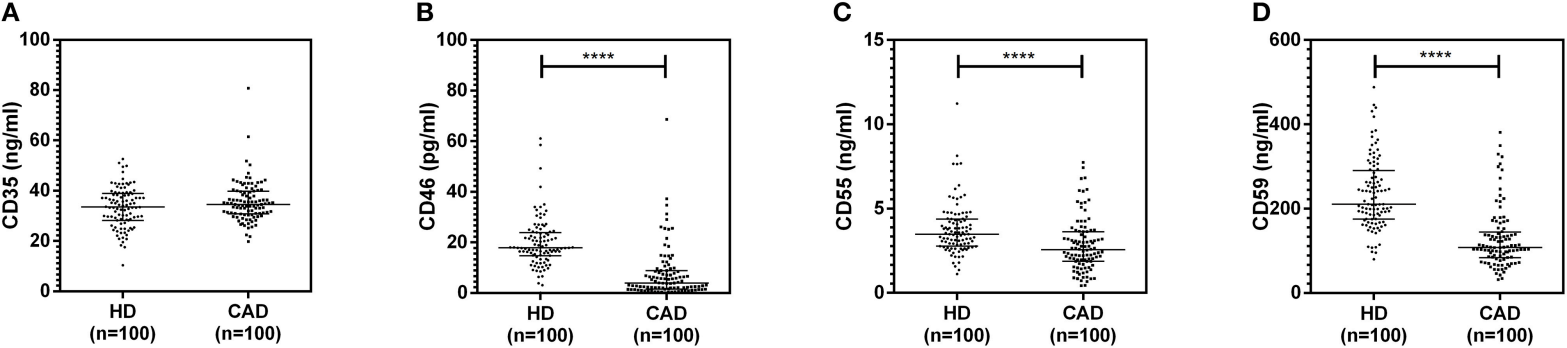
Plasma levels of soluble forms of complement regulatory proteins in CAD patients. Soluble forms of CD35, CD46, CD55, and CD59 in the plasma were measured by sandwich ELISA **(A–D)**. Plasma levels of CD46, CD55, and CD59 were significantly lower in CAD patients compared to healthy donors. Mann-Whitney *U* test was used for comparison of values between groups. Four asterisks (****) indicate a *p*-value of <0.001.

**Figure 5:**
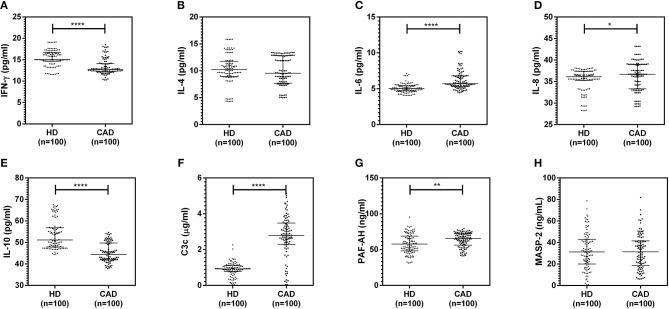
Inflammatory and complement activation status in CAD patients **(A–H)**. Plasma levels of IL-4, IL-6, IL-8, IL-10, IFN-γ, C3c, and PAF-AH were measured by sandwich ELISA. Significantly higher complement activation, as assessed by C3c levels, IL-6, IL-8, and PAF-AH levels were observed in CAD patients compared to healthy donors. IFN-γ and IL-10 levels were significantly lower in CAD patients compared to healthy donors. Mann-Whitney *U* test was used for comparison of values between groups. One asterisk (*) indicates a *p*-value of <0.05, two asterisks (**) indicate a *p*-value of <0.01, and four asterisks (****) indicate a *p*-value of <0.001.

**Figure 6:**
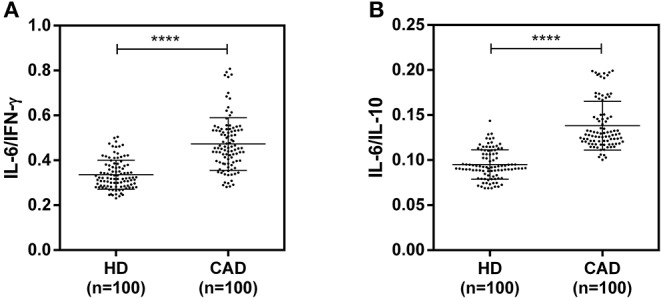
Chronic inflammation status in CAD patients **(A,B)**. Ratios of IL6/IFN-γ and IL6/IL10 in CAD patients were calculated and used as a reference for the status of chronic inflammation. Mann-Whitney *U* test was used for comparison of values between groups. Four asterisks (****) indicate a *p*-value of <0.001.

The plasma levels of soluble forms of CD35 were unaltered, while levels of CD46, CD55, and CD59 were significantly lower in CAD patients compared to healthy donors (p<0.0001) (Figure 4). The plasma levels of soluble Cregs were not significantly different between CAD patients with SVD, DVD or TVD (data not shown). The plasma levels of cytokines IL-4, IL-6, IL-8, IL-10, and IFN-γ were assessed as markers of inflammatory status. Soluble levels of PAF-AH were used as independent markers of CAD. C3c, the final split product of complement protein C3, was measured to assess the complement activation status. The plasma levels of IFN-γ, and IL-10 were significantly lower in CAD patients as compared to healthy donors (*p* < 0.0001). The levels of IL-6, IL-8, C3c, and PAF-AH were significantly higher in CAD patients as compared to the healthy donors (*p* < 0.0001). The plasma levels of IL-4 and MASP-2 were comparable between CAD patients and healthy controls (Figure 5). The ratio of IL-6/IFN-γ and that of IL-6/IL-10, measures of chronic inflammation, were significantly higher in CAD patients (*p* < 0.0001) than healthy donors (Figure 6). No significant difference was observed in the levels of plasma cytokines in the CAD subgroups (data not shown). Taken together, the cytokine profiling was suggestive of an overall chronic immune and complement activation.

## Discussion

Membrane complement regulatory proteins are expressed widely on different cell populations. Dysregulation of the Cregs, proteins responsible for maintaining homeostasis of the complement cascade, has been implicated in several autoimmune disorders, chronic inflammatory diseases, diabetes, cancers and malaria (11, 16, 18, 24, 25). Unabated activation of the complement cascade and increased expression of inflammatory cytokines contribute significantly to the immune inflammatory manifestations in CAD (4, 26, 27). Limited information is available on the regulation of the complement cascade in CAD. Therefore, in this study, we evaluated the expression of complement regulatory proteins CD35, CD46, CD55, and CD59 on granulocytes, lymphocytes, and monocytes of CAD patients and healthy donors. Simultaneously, the levels of selected cytokines and soluble forms of these Cregs in plasma were determined to gain a comprehensive insight into the inflammatory milieu in CAD.

Among the Cregs analyzed on the surface of lymphocytes, monocytes and granulocytes of healthy donors and CAD patients, significantly lower expression of CD46 and CD55 was observed on all leukocyte subsets in CAD patients suggesting a global downregulation of these two Cregs in CAD patients, irrespective of disease stage. Both these proteins inhibit the complement cascade at the level of C3; CD46 by facilitating C3 degradation and CD55 by indirectly dissociating the C3 convertases (7, 8), thus preventing generation of inflammatory peptides and membrane attack complexs downstream and protecting the host from complement-mediated injury. It is plausible that this could have aggravated the complement activation and related pathological manifestations in patients with CAD. A disease related decline in Cregs has earlier been reported in Rheumatoid arthritis (RA) (24, 25). Herein, we observed a significantly high surface expression of CD35 on granulocytes and of CD59 on lymphocytes and granulocytes of CAD patients. Furthermore, CD59 surface expression on granulocytes was significantly higher in multivessel blockage than in single vessel blockage, suggesting a positive association with increasing vessel blockage. This may be confirmed by carrying out a longitudinal study to evaluate the alterations in CD59 expression at different disease stages of CAD. A deficiency of CD59 in mice models has been shown to accelerate the development of atherosclerotic plaques, suggesting a protective role of CD59 in atherosclerosis (28, 29). Activation of neutrophils, the major constituent of granulocytes, has been shown to increase the surface expression of CD59 (30). Increased CD59 expression on cells leads to sublytic doses of MAC and thereby an increased resistance to complement-mediated cell damage, favoring cell survival (12, 27, 31). The complement regulatory role of CD35 is by acting as a cofactor for factor I mediated cleavage of C3b and C4b while CD59 acts at the last step of the complement cascade and inhibits the formation of membrane attack complex (8). It can be speculated that the high expression of CD35 and CD59 observed on granulocytes is perhaps a preventive measure against the generation of inflammatory peptides and MAC in an attempt toward conferring protection to the host with CAD from complement mediated injury. The modulation of the downstream signaling mechanisms mediated by high expression of CD35 and CD59 on the respective immune cells in the CAD patients have not been addressed and is a limitation of this study.

We next assessed the mRNA expression of CD35, CD46, CD55, and CD59 by quantitative PCR. Except for CD35, the mRNA expression of CD46, CD55, and CD59 did not vary between the study groups. Unaltered mRNA expression of CD46, CD55, and CD59 with their differential surface protein expression (lower expression for CD46 and CD55, and higher expression for CD59) in CAD patients may perhaps be due to alterations in regulation at the steps of protein translation or post-translational modifications, although it remains to be addressed in detail. In SLE and RA, reduced transcript levels of CD35 mRNA have been documented, and correlated negatively with complement activation (32, 33). In neutrophils, the major constituent of granulocytes, CD35 molecules are present as cytoplasmic secretory vesicles that are translocated to plasma membrane on cell activation (34), and serve as key receptor for phagocytosis. In our study, we observed significantly higher surface expression of CD35 on granulocytes, and though no correlation was observed between CD35 transcript and CD35 surface expression of granulocytes, it can be hypothesized that CD35 surface expression on granulocytes can plausibly be modulated at the transcript level. However, the mRNA expression profile in this study was done at the level of total leukocytes and the exact association between mRNA and surface expression of Cregs within leukocyte subsets can only be confirmed by assessing the mRNA expression within individual lymphocytic, monocytic, and granulocytic clusters.

The soluble forms of CD35, CD46, CD55, and CD59 are released from the blood cells, primarily leukocytes, via different processes including cell damage and activation, cleavage by matrix metalloproteinases (MMPs), enzymatic cleavage via phospholipase C and/or D, and have been implicated in several chronic inflammatory diseases including SLE, Chagas disease, and diabetic retinopathy (12–14, 23). Here we observed that, with the exception of CD35, the plasma levels of soluble forms of CD46, CD55, and CD59 were significantly lower in CAD patients, though no difference was seen with varying disease severity.

The inflammatory milieu of CAD is to a great extent contributed by cytokines and active complement fragments (2–4). Therefore, we assessed the plasma levels of cytokines IL-4, IL-6, IL-8, IL-10, and IFN-γ as markers of inflammatory status. Soluble levels of PAF-AH were used as an independent marker of CAD (35). C3c, the final split product of complement protein C3, was measured to assess complement activation status (36). Levels of IL-6, IL-8, IL-10, IFN- γ, C3c, and PAF-AH were significantly altered in CAD patients compared to healthy donors. The ratios of IL-6/IFN-γ and IL-6/IL-10, a measure of chronic inflammation (37, 38), were significantly higher in CAD patients though no significant difference was observed in the levels of plasma cytokines in the CAD subgroups. Although there was no significant correlation between the cytokine levels and relative surface protein expression of Cregs; the cytokine profile and high levels of C3c were indicative of a chronic inflammatory state in the CAD patients. High levels of IL-6, IL-8, and low IL-10 levels have also been documented in previous studies conducted in CAD patients (39–41). A similar trend of higher IL-6 levels, with lower IL-10 and IFN-γ, has been observed in RA patients and attributed to chronic inflammation (32).

MASP-2, one of the serine proteases responsible for the activation of the lectin pathway, was recently implicated as an important mediator of ischemia/reperfusion injury in animal models and was shown to be significantly reduced in patients suffering from myocardial infarction, and anti-MASP-2 antibodies were shown to significantly reduce the risk of ischemic damage (26, 42). In this study population, we, however, did not observe any significant difference between the levels of MASP-2 in the peripheral circulation of CAD patients and their subgroups and healthy controls.

The CAD patients included in this study were all on statin therapy, however, we have not evaluated whether the expression of Cregs on the leucocytes of CAD patients was altered by statins and is a limitation that needs to be addressed. Statins reduce cholesterol biosynthesis by inhibiting the HMG-CoA reductase, the rate-limiting enzyme in cholesterol biosynthesis, and are widely prescribed to reduce atherosclerotic complications due to hyperlipidemia (43). In addition, *in vitro* studies have shown that statins induce increased expression of CD55 and CD59 on the vascular endothelium (44, 45). Moreover, statins have been shown to exert additional protective actions, independent of their lipid lowering ability. Several studies conducted in animal models have shown the immunomodulatory roles of statins such as a reduction in the secretory levels of IL-6, TNF-α, IL-1β, IL-8, and MCP-1 (46–48). However, in majority of these studies, statins have been used at concentrations that far exceed those achieved in the human plasma in pharmacokinetic studies (49). Evaluation of the expression profile of Cregs in this study was limited to the monocytic, lymphocytic, and granulocytic clusters. More information could be gained if we had assessed the expression of Cregs on the individual cell lineages of T cells, B cells, dendritic cells and neutrophils among others to delineate the precise role of Cregs in modulating the immune response in CAD. Another shortcoming of this study is that the majority of the CAD patients and controls enrolled were male subjects. Due to the cross-sectional nature of this study and plausibly because of the recruitment being done from a tertiary care hospital and the willingness of the patients to participate in the study, our study population showed a profound gender bias toward the male population. Healthy donors were gender-matched with the CAD patients for this study and therefore the controls were also predominantly males.

To summarize, a significantly lower expression of CD46 and CD55 on all leukocyte subsets and a significantly higher expression of CD59 on granulocytes and lymphocytes and that of CD35 on granulocytes was observed in statin-treated CAD patients as compared to healthy individuals. While the extent of modulation of the Cregs expression remained comparable at the different disease stages of the CAD patients for different parameters, a differential and significantly high CD59 expression was seen on granulocytes in the order of SVD < DVD < TVD. The findings of this study suggest that CD59 may perhaps serve as a putative marker in the assessment of the severity of CAD, that needs to be assessed by undertaking longitudinal cohort studies in CAD patients.

## Data Availability

The datasets generated for this study are available on request to the corresponding author.

## Author Contributions

NM and MM performed the experiments. NM wrote the manuscript and analyzed the data with the help of RP. RN provided the CAD samples and clinically managed the patients. RL performed the lipid analysis. AH provided the healthy donor samples. ND conceived the study. KL coordinated and monitored the work execution and analysis, edited, and finalized the manuscript along with ND and with inputs from RN and RL.

### Conflict of Interest Statement

The authors declare that the research was conducted in the absence of any commercial or financial relationships that could be construed as a potential conflict of interest.
